# The effect of pessary treatment on puborectalis muscle function

**DOI:** 10.1007/s00192-021-04766-2

**Published:** 2021-04-13

**Authors:** Claudia Manzini, Frieda van den Noort, Anique T. M. Grob, Mariëlla I. J. Withagen, Carl H. van der Vaart

**Affiliations:** 1 Department of Reproductive Medicine and Gynecology, University Medical Center Utrecht, Utrecht, The Netherlands; 2 Robotics and Mechatronics, Faculty of electrical engineering mathematics and computer science, Technical Medical Centre, University of Twente, Enschede, The Netherlands; 3 Multimodality Medical Imaging Group, Faculty of Science and Technology, Technical Medical Centre, University of Twente, Enschede, The Netherlands

**Keywords:** Pelvic organ prolapse, Vaginal pessaries, Puborectalis muscle, Pelvic floor, Avulsion, Transperineal ultrasound

## Abstract

**Introduction and hypothesis:**

The objective was to assess if puborectalis muscle (PRM) function changes in women with pelvic organ prolapse (POP) undergoing pessary treatment.

**Methods:**

This was a prospective cohort study of women with symptomatic POP choosing pessary treatment. An interview, clinical examination and 3D/4D transperineal ultrasound were performed at baseline and at 3-month follow-up. POP was assessed using the Pelvic Organ Prolapse Quantification system (POPQ). Parameters compared between baseline and follow-up were: hiatal area at rest (HArest), maximal contraction (HActx), and maximal Valsalva maneuver (HAVal), displacement in contraction (DISPL-ctx, i.e., relative difference between HArest and HActx), and displacement in Valsalva (DISPL-Val, i.e., relative difference between and HAVal and HArest). Parameters were compared in women with and those without complete avulsion.

**Results:**

A total of 162 women were assessed and 34 were included. Mean age was 64 years (SD 11.4), and mean BMI 24 kg/m^2^ (SD 3.1). Thirty-one women had a cystocele, 8 a uterine prolapse, and 12 had a posterior compartment prolapse. Twenty-one women (61.8%) had a POP stage II, and 13 (38.2%) a POP stage III. Ring pessaries were most frequently used (97%). In the entire group a statistically significant increase in DISPL-ctx was observed (mean difference 2.1%, *p* = 0.017). In the no avulsion group HArest and DISPL-ctx increased significantly (mean difference 4.1%, *p* = 0.016 and 2.7%, *p* = 0.016 respectively) and the increase in DISPL-ctx was higher than in the avulsion group (mean difference 2.7% vs 0.2%, *p* = 0.056).

**Conclusion:**

Our results show that PRM function changes in women with POP undergoing pessary treatment and suggest that such change occurs mainly in the absence of complete avulsion.

## Introduction

The levator ani muscle (LAM) plays a crucial role in the pathophysiology of pelvic organ prolapse (POP) [1, 2]. Under normal conditions, the LAM tightens the levator hiatus (i.e., the area encircled by the pubic bone and LAM) and provides a lifting force, making the pelvis an isobaric chamber [3]. One of the current theories of POP development [3] proposes that, if the LAM is damaged, the levator hiatus is widened and the vagina becomes exposed to the pressure differential between abdominal and atmospheric pressures. As a consequence, a pressure gradient arises in the pelvis, and the pelvic organs descend. On transperineal ultrasound (TPUS) the levator hiatus can be visualized as the area encircled by the puborectalis muscle (PRM, one of the LAM subdivisions) and the pubic bone. TPUS studies confirmed the association between enlarged levator hiatus and POP [4]. Furthermore, computer simulation studies showed the role of an increased hiatus size (defined as the distance between pubic symphysis to the ventral tip of the perineal body) in the development of POP [5]. Given the crucial role of the LAM in POP pathophysiology, treatments aimed at improving LAM function, such as pelvic floor muscle treatment (PFMT), are beneficial [6].

Pessary treatment is the other conservative option for POP [7, 8] and has proven effective in relieving POP symptoms by physically supporting the vaginal walls and the pelvic organs behind them [9–12]. Our hypothesis is that pessary treatment, by supporting the vaginal walls and the pelvic organs, counteracts the abnormal pressure gradient that has arisen during POP development. In this way, the pressure the LAM is exposed to could be reduced and the LAM, or some of its subdivisions (such as the PRM), could partially regain their function as the result of tissue remodeling or a physical effect [13].

Evidence in this respect is limited. Jones and coworkers observed a decrease in genital hiatus size (i.e., GH of the Pelvic Organ Prolapse Quantification system, POPQ) after 3 months of pessary use. They concluded that pessary use may result in some degree of LAM recovery [13]. However, genital hiatus size only provides an indirect assessment of the LAM. In order to determine the status of the LAM, it has to be visualized using imaging techniques. The aim of our study is to investigate with TPUS if an average of 3 months of pessary treatment is associated with changes in PRM function. We refer to PRM function instead of LAM function, because, as mentioned before, the PRM is the LAM subdivision surrounding the levator hiatus as assessed on TPUS. In addition, we analyzed the influence of avulsion on the change in PRM function observed during pessary treatment.

## Materials and methods

The data used in the current study were collected as a subset within the GYNecological Imaging using 3D UltraSound (GYNIUS) project on the assessment of pelvic floor contractility with TPUS, which was conducted at our urogynecological center, where secondary and tertiary care are provided. Women were included in the GYNIUS project between May 2018 and December 2019. The Medical Research Ethics Committee (MREC) exempted the project from ethical approval (reference 18/215), because TPUS was part of our routine diagnostic procedures and standard care. All women signed informed consent forms.

This was a prospective cohort study. Inclusion criteria were: women with symptomatic POP choosing pessary treatment, and successful pessary use during the study period. Exclusion criteria were: women already using a pessary at baseline; pessary fitting started more than 4 weeks after baseline assessment; women not attending the 3-month follow-up at our clinic; women undergoing pelvic floor muscle training (PFMT) in combination with pessary treatment during the study period. POP stage was not an inclusion/exclusion criterium. The rationale of the second exclusion criterium (i.e., pessary fitting started more than 4 weeks after baseline assessment) was the following. In the case of a long period between baseline TPUS and the start of pessary fitting, the baseline PRM function could have been unreliable because the hiatal dimensions might have changed in the meantime for reasons other than pessary treatment. To avoid this possible confounder, a maximum of 4 weeks between baseline assessment and start of pessary fitting was accepted.

At baseline and regular follow-up, all women underwent an interview, clinical examination, and 3D/4D TPUS. POP was assessed using the Pelvic Organ Prolapse Quantification system (POPQ) [14]. At baseline, pessary fitting was performed according to our standard clinical practice, similar to that described in the literature [15–20]. Based on clinical examination, a ring pessary of appropriate size (without or with support) was inserted at the initial fitting. If a ring pessary was not suitable, Gellhorn, donut or cube pessaries were tried. The following appointment was scheduled after 2–4 weeks to assess if the first pessary fitting trial was successful. A fitting trial was considered successful if the woman decided to continue using the pessary she was fitted with. If not, a different pessary size or type could be tried, and another pessary fitting trial was performed. This process was repeated until a successful fitting was achieved or pessary treatment was considered not suitable for the woman. The follow-up for pessary management and repeated TPUS was scheduled 3 months after successful pessary fitting. The choice of having the second assessment 3 months after successful pessary fitting was based on the study of Jones and coworkers [13] (in which the change in genital hiatus size was assessed 3 months after pessary use) and on convenience because our standard clinical practice consists of a follow-up 3 months after successful pessary fitting.

The TPUS was performed in supine position with an empty bladder. Women were instructed to perform maximal pelvic floor contraction and maximal Valsalva maneuver according to the method described by Dietz [21]. We used a Philips Epiq 7G machine with a X6–1 transducer covered with a 2 cm thick gel pad, and a glove. The gel pad was used to create more distance between the transducer and the women, so that the LAM could be fully visible within the opening angle on the coronal plane. TPUS volumes analyzed in the current study were acquired without pessary in situ. At follow-up the pessary was removed around 20 min before performing the TPUS.

Transperineal ultrasound volumes were assessed by the first author, using a tool developed by the second author in the image processing software MeVisLab [22]. This tool enables the selection of the correct frame and plane and the assessment of levator hiatal areas and avulsion. The first author was blinded against all clinical data and did not know which TPUS was acquired at baseline and which one at follow-up. As described in the literature [23], hiatal area at rest (HArest), on maximal pelvic floor contraction (HActx), and on maximal Valsalva maneuver (HAVal) were manually segmented at the plane of minimal hiatal dimensions (Appendix 1). If a woman could not perform pelvic floor contraction, HArest was also used for HActx. In Table 1 the parameters derived per woman from the manual segmentations are listed.

**Table 1:** Parameters derived per woman (i) from the manually segmented hiatal dimensions

Parameter	Formula
DISPL-ctx_i_	(HArest_i_ - HActx_i_)/HArest_i_
DISPL-Val_i_	(HAVal_i_ - HArest_i_)/HArest_i_
Δ HArest_i_	(HArest at follow-up_i_ – HArest at baseline_i_)/HArest at baseline_i_
Δ HActx_i_	(HActx at follow-up_i_ – HActx at baseline_i_)/HActx at baseline_i_
Δ HAVal_i_	(HAVal at follow-up_i_ – HAVal at baseline_i_)/HAVal at baseline_i_
Δ DISPL-ctx_i_	DISPL-ctx at follow-up_i_ – DISPL-ctx at baseline_i_
Δ DISPL-Val_i_	DISPL-Val at follow-up_i_ – DISPL-Val at baseline_i_

*DISPL-ctx* displacement in contraction, *DISPL-Val* displacement in Valsalva, *HArest* hiatal area at rest, *HActx* hiatal area on maximal pelvic floor contraction, *HAVal* hiatal area on maximal Valsalva maneuver

After having segmented HArest, HActx, and HAVal, the presence of avulsion was assessed at a later stage by the first author on baseline volumes obtained at maximum contraction. The assessor was blinded against all levator HA measurements while performing avulsion assessment. On tomographic imaging (TUI) a 2.5-mm interslice interval was set. The central slice was placed at the plane of minimal hiatal dimensions, showing the symphysis pubis closing medially. Complete avulsion was defined as a levator–urethra gap of ≥25 mm on the three central slices on the right side, on the left side (unilateral) or both sides (bilateral), as shown in Appendix 2 [23]. Avulsion was defined based on the presence of complete unilateral or bilateral avulsion.

To the best of our knowledge, TPUS parameters have never been used to assess the effect of pessary treatment on PRM function. Therefore, no formal sample size could be calculated, and this work can be considered an exploratory study.

Our primary outcome was to assess if the deltas (i.e., relative differences between follow-up and baseline calculated per woman) were significantly different from zero in the entire group, and if parameters were significantly different between the avulsion group and no-avulsion group. A *t* test was performed in the case of normally distributed data, as assessed by the Shapiro–Wilk test (*p* > 0.05), and if there were no outliers in the data. Otherwise, a one-sample Wilcoxon signed rank test or an independent samples Mann–Whitney *U* test was run. The effect size was calculated using Cohen’s d, when appropriate [24]. The statistical analysis was conducted using IBM v 27 SPSS software.

## Results

**Fig. 1:**
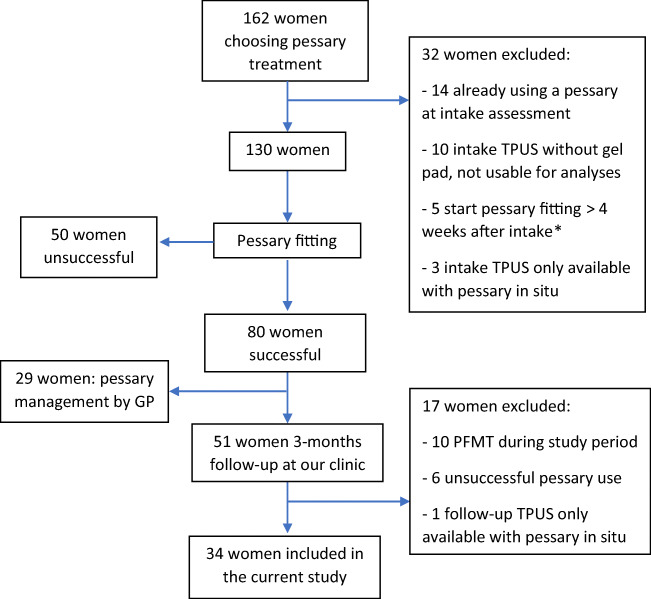
Number of women at each stage. *Of these 5 women 3 had an additional exclusion criterium: 2 did not attend the follow-up at our clinic (1 attended it at the GP clinic and the other had a telephone appointment because of the COVID-19 pandemic) and 1 underwent PFMT. The 2 women who were excluded only based on this criterium had an intake assessment to pessary fitting interval ≥12 weeks. *TPUS* transperineal ultrasound, *GP* general practitioner, *PFMT* pelvic floor muscle treatment

Figure 1 shows the number of women at each stage. Initially, 162 women choosing pessary treatment were included in the GYNIUS project. Inclusion and exclusion criteria left 34 women to be included in the current study.

Mean age was 64 years (SD 11.4), and mean BMI 24 kg/m^2^ (SD 3.1). Thirty (88%) women were postmenopausal, and 32 (94%) vaginally parous with only one vacuum-extraction and one forceps delivery. Ten (29%) women had undergone prior gynecological surgeries, i.e., 3 vaginal hysterectomies, 3 abdominal hysterectomies, 2 anterior repairs, 2 posterior repairs, 1 sacrospinous fixation, and 1 POP surgery not specified. On clinical examination 31 (91%) had a significant (POPQ ≥2) cystocele, 8 (24%) a uterine prolapse, and 12 (35%) a posterior compartment prolapse. Twenty-one women (61.8%) had a POP stage II, and 13 (38.2%) had a POP stage III. For 19 women (56%) pessary fitting was successful at the first trial, whereas 15 women (44%) needed adjustment of the pessary size or type before being successful. Thirty-three (97%) were successfully fitted with a ring pessary (without or with support), and 1 (3%) with a Gellhorn pessary. The second TPUS was performed an average of 3.5 months (SD 1.1) after the insertion of the successful pessary.

Table 2 shows median and interquartile range (IQR) of HArest, HActx, HAVal, DISPL-ctx, and DISPL-Val at baseline and follow-up. One woman was unable to perform pelvic floor contractions. Therefore, HArest was also used for HActx.

**Table 2:** Median and interquartile range of hiatal area at rest (HArest), maximal pelvic floor contraction (HActx) and maximal Valsalva maneuver (HAVal), displacement in contraction (DISPL-ctx), and displacement in Valsalva (DISPL-Val) at baseline and follow-up

Parameter	Baseline (*n* = 34), median (IQR)	Follow-up (*n* = 34), median (IQR)
HArest (cm^2^)	19.8 (4.7)	20.2 (5.9)
HActx (cm^2^)	16.7 (4.2)	16.3 (4.6)
HAVal (cm^2^)	30.6 (13.5)	31.8 (9.5)
DISPL-ctx (%)	17.2 (14.0)	19.0 (19.0)
DISPL-Val (%)	50.7 (45.0)	52.9 (40.0)

*DISPL-ctx* (HArest – Hactx)/HArest, *DISPL-Val* (HAVal – HArest)/HArest

Table 3 shows the results of a one-sample *t* test assessing the relative difference between follow-up and baseline of Δ HArest, Δ HActx, Δ DISPL-ctx, and Δ DISPL-Val. DISPL-ctx increased significantly from baseline to follow-up. On a one-sample Wilcoxon signed rank test the median of Δ HAVal was not significantly different from zero (median (IQR) 3.8 (16), *p* = 0.14).

**Table 3:** Results of a one-sample *t* test (test value: 0) assessing the relative difference between follow-up and baseline (*n* = 34)

Parameter	Mean difference (SD) %	*p* value	95% CI (%)	
Δ HArest	3.0 (9.3)	0.073	−0.3	6.2
Δ HActx	−0.1 (7.6)	0.910	−2.8	2.5
Δ DISPL-ctx	2.1 (4.9)	**0.017**	0.4	3.8
Δ DISPL-Val	0.6 (18.0)	0.836	−5.6	6.9

*Δ HArest* (HArest at follow-up – HArest at baseline)/HArest at baseline, *Δ HActx* (HActx at follow-up – HActx at baseline)/HActx at baseline, *Δ DISPL-ctx* DISPL-ctx at follow-up – DISPL-ctx at baseline, *Δ DISPL-Val* DISPL-Val at follow-up – DISPL-Val at baseline

Parameters of the avulsion and no-avulsion groups were compared. The results of this analysis are reported in Table 4. In addition, the deltas were assessed in the two groups, separately (Table 5). In the no-avulsion group HArest and DISPL-ctx increased significantly from baseline to follow-up with an effect size of 0.51 and 0.50 respectively, whereas the median of Δ HAVal was not significantly different from zero on a one-sample Wilcoxon signed rank test (median (IQR) −5.5 (16.0), *p* = 0.086).

**Table 4:** Comparison of the avulsion group and no-avulsion group (independent samples *t* test if not otherwise specified)

Parameter	No-avulsion group (*n* = 26)	Avulsion group (*n* = 8)	*p* value
HArest at baseline, median (IQR)	19.8 (4.5)	19.7 (10.0)	0.436*
HArest at follow-up, mean (SD)	20.6 (3.5)	22.1 (6.4)	0.387
Δ HArest, mean (SD) %	4.1 (8.0)	−0.6 (12.6)	0.351
HActx at baseline, median (IQR)	16.1 (3.9)	17.4 (7.8)	0.077*
HActx at follow-up, mean (SD)	15.8 (2.9)	19.2 (4.8)	**0.020**
Δ HActx, mean (SD) %	0.1 (6.5)	−1.0 (11.1)	0.806
HAVal at baseline, median (IQR)	30.3 (13.2)	35.6 (13.5)	0.253*
HAVal at follow-up, median (IQR)	29.3 (9.3)	34.8 (14.5)	0.327*
Δ HAVal, median (IQR) %	5.5 (16.0)	−0.9 (11.0)	0.327*
DISPL-ctx at intake, mean (SD) %	19.9 (10.0)	11.7 (10.1)	**0.049**
DISPL-ctx at follow-up, mean (SD) %	22.6 (11.7)	11.8 (10.3)	**0.025**
Δ DISPL-ctx, mean (SD) %	2.7 (5.4)	0.2 (2.0)	0.056
DISPL-Val at intake, median (IQR) %	50.7 (46.0)	51.6 (36.0)	0.618*
DISPL-Val at follow-up, mean (SD) %	54.2 (29.0)	55.2 (17.7)	0.923
Δ DISPL-Val, mean (SD) %	0.7 (18.9)	0.5 (15.5)	0.979

*DISPL-ctx* (HArest – Hactx)/HArest, *DISPL-Val* (HAVal – HArest)/HArest, *Δ DISPL-ctx* DISPL-ctx at follow-up – DISPL-ctx at baseline, *Δ DISPL-Val* DISPL-Val at follow-up – DISPL-Val at baseline

Bold indicates the significant parameters

*Independent samples Mann–Whitney *U* test

**Table 5:** Results of a one-sample *t* test (test value: 0) assessing the relative difference between follow-up and baseline in the no-avulsion group and the avulsion group, separately

Group	Parameter	Mean difference (SD) %	*p* value	95% CI (%)	
No-avulsion (*n* = 26)	Δ HArest	4.1 (8.0)	**0.016**	0.8	7.3
	Δ HActx	0.1 (6.5)	0.940	−2.5	2.7
	Δ DISPL-ctx	2.7 (5.4)	**0.016**	0.5	4.9
	Δ DISPL-Val	0.7 (18.9)	0.855	−7.0	8.3
Avulsion (*n* = 8)	Δ HArest	−0.6 (12.6)	0.894	−11.2	10.0
	Δ HActx	−0.9 (11.1)	0.816	−10.2	8.3
	Δ HAVal	−0.4 (7.6)	0.891	−6.7	5.9
	Δ DISPL-ctx	0.2 (2.0)	0.792	−1.5	1.9
	Δ DISPL-Val	0.5 (15.5)	0.931	−12.5	13.5

Bold indicates the significant parameters

There was no difference in the deltas between women with POP stage II and women with POP stage III.

## Discussion

A statistically significant increase in DISPL-ctx was observed 3 months after successful pessary fitting. This result is consistent with the hypothesis that pessary treatment is associated with changes in PRM function. Moreover, in the no-avulsion group HArest and DISPL-ctx increased significantly and the increase in DISPL-ctx was higher than in the avulsion group (*p* = 0.056).

DISPL-ctx can increase from baseline to follow-up as a result of a decrease in HActx, an increase in HArest, or both. We found a very small, nonsignificant decrease in HActx, whereas HArest increased (*p* = 0.07). This implies that the increase in DISPL-ctx was more driven by an increase in HArest than by a decrease in HActx, which is also confirmed by the statistically significant increase in HArest in the no-avulsion group. Whether the changes observed can be interpreted as a regain of PRM function or not is questionable. A possible explanation for these findings is that women with POP try to relieve their POP symptoms by contracting the PRM, which counteracts the abnormal pressure gradient originating during POP development. Vaginal pessaries, by supporting POP, could reduce the need for this continuous contraction, allowing the PRM to relax (which was measured as an increase in HArest). From this perspective, the increase in DISPL-ctx is the result of a more physiological resting position. In the following, we refer to this explanation of our results as the “contraction hypothesis.” An alternative explanation is that a progressive relaxation of the resting tone occurs in women with POP undergoing pessary treatment, which can be clinically experienced by the need for a bigger pessary size after some time of pessary use. In the following, we refer to this alternative explanation as the “relaxation hypothesis.” The difference between the two hypotheses lies in the baseline resting tone of the PRM, which is not fully relaxed in the “contraction hypothesis,” whereas it is fully relaxed in the “relaxation hypothesis.”

At baseline and follow-up, women with complete avulsion had significantly lower DISPL-ctx than those in the no-avulsion group, which confirms previous results [25]. Moreover, no significant change in DISPL-ctx was observed during pessary treatment in the avulsion group, whereas a significant increase was observed in the no-avulsion group (with a medium effect size). The difference in DISPL-ctx between the two groups was almost significant (*p* = 0.056). These findings are more consistent with the “contraction hypothesis” and can be explained by the impaired ability to contract of women with complete LAM avulsion. These results are more difficult to explain with the “relaxation hypothesis” because a higher relaxation of the resting tone over time can be expected in the case of a damaged muscle.

A few studies investigated whether pessary treatment has an effect on pelvic floor anatomical parameters. Jones and coworkers compared the genital hiatus size of 42 women at baseline and after 3 months of pessary use [13]. They observed a decrease in genital hiatus size at rest and in Valsalva, with the greatest change registered in women using a Gellhorn pessary. There are several possible explanations for the discrepancy between their results and ours. First, we included only women who did not undergo PFMT, whereas they did not specify if this selection was made (and PFMT has proven to be associated with a reduction in HArest in women with POP [26]). Second, a larger proportion of women used a Gellhorn pessary in their study, and the greatest change in genital hiatus was registered in this subgroup. Third, genital hiatus and levator HA on TPUS are different measurements: genital hiatus is the distance between the middle of the external urethral meatus and the posterior margin of the hymen, whereas the levator HA on TPUS is the area encircled by the pubic bone and PRM. Therefore, they could reflect the function of different pelvic floor muscles (i.e., the puboperineal muscle and the PRM respectively [3]). Fourth, TPUS allows for the visualization and thus for a better assessment of the pelvic floor muscles compared with clinical examination. Last, we observed a significant increase in DISPL-ctx, which they did not assess.

Lone and coworkers evaluated levator hiatus dimensions using 3D endovaginal ultrasound before and 1 year after surgery, no treatment, or pessary treatment for POP [27]. No change was observed after pessary treatment. However, DISPL-ctx was not assessed in their study because only dimensions at rest can be measured with endovaginal ultrasound. They did not assess women with and without avulsion separately. Moreover, only 6 scans of the 10 women undergoing pessary treatment were analyzable at follow-up. Therefore, a significant change was unlikely to be measured in this group.

Our study has several strengths. First, all scans were performed by the same clinician, thus reducing a source of variability. Second, the assessor was blinded to all clinical data and did not know which TPUS was acquired at baseline and follow-up. Intra-observer variability is not expected to introduce a bias in levator HA measurements, as their repeatability has been proven to be very high [28, 29]. Third, the assessor was blinded against all levator HA measurements while performing avulsion assessment. Fourth, to eliminate a possible confounder, only women who did not undergo PFMT were included. Although we cannot exclude that women performed pelvic floor exercises by themselves, none had supervised PFMT and at follow-up all denied having exercised themselves.

Some limitations must also be acknowledged. We did not have a control group. Therefore, it cannot be excluded that the changes we observed reflect the natural course of POP. However, we measured a statistically significant increase in DISPL-ctx and HArest (in the no-avulsion group) in a relatively small sample and in a short period of time, which is unlikely to be observed in women who do not undergo any treatment. The changes we observed were statistically significant but relatively small. Therefore, their clinical significance has to be further investigated in larger studies. In addition, the size of the avulsion group might have limited the detection of significant changes in this group. However, the differences between avulsion group and no-avulsion group are clear. A 3-month follow-up might have been short to fully appreciate the effect of pessary treatment on PRM function and future studies with a long-term follow-up should be performed. An additional limitation is the relatively large proportion of dropouts, which might have introduced a selection bias. Last, our results may not be extended to all women with POP successfully fitted with any type of vaginal pessary: the study was conducted in a urogynecological center (where primary care is not provided) and the majority of women were fitted with a ring pessary.

Being aware of these limitations, the results of our exploratory study can stimulate future research. Women without avulsion can have a normally functioning, underactive or overactive pelvic floor. It would be interesting to compare the effect of pessary treatment on PRM function between these groups. One randomized control trial showed the benefit of adding pessary treatment to PFMT for POP symptoms improvement [30]. If the “contraction hypothesis” is correct (i.e., if pessary treatment enables the PRM to fully relax at rest), pessary treatment might also allow for a better PRM function improvement in women undergoing PFMT. Our study provides an outcome measure (i.e., DISPL-ctx) that can be used to test this hypothesis.

In conclusion, our results show that PRM function changes in women with POP undergoing pessary treatment and suggest that such change occurs mainly in the absence of complete avulsion.

## Figures and Tables

**Fig. 2:**
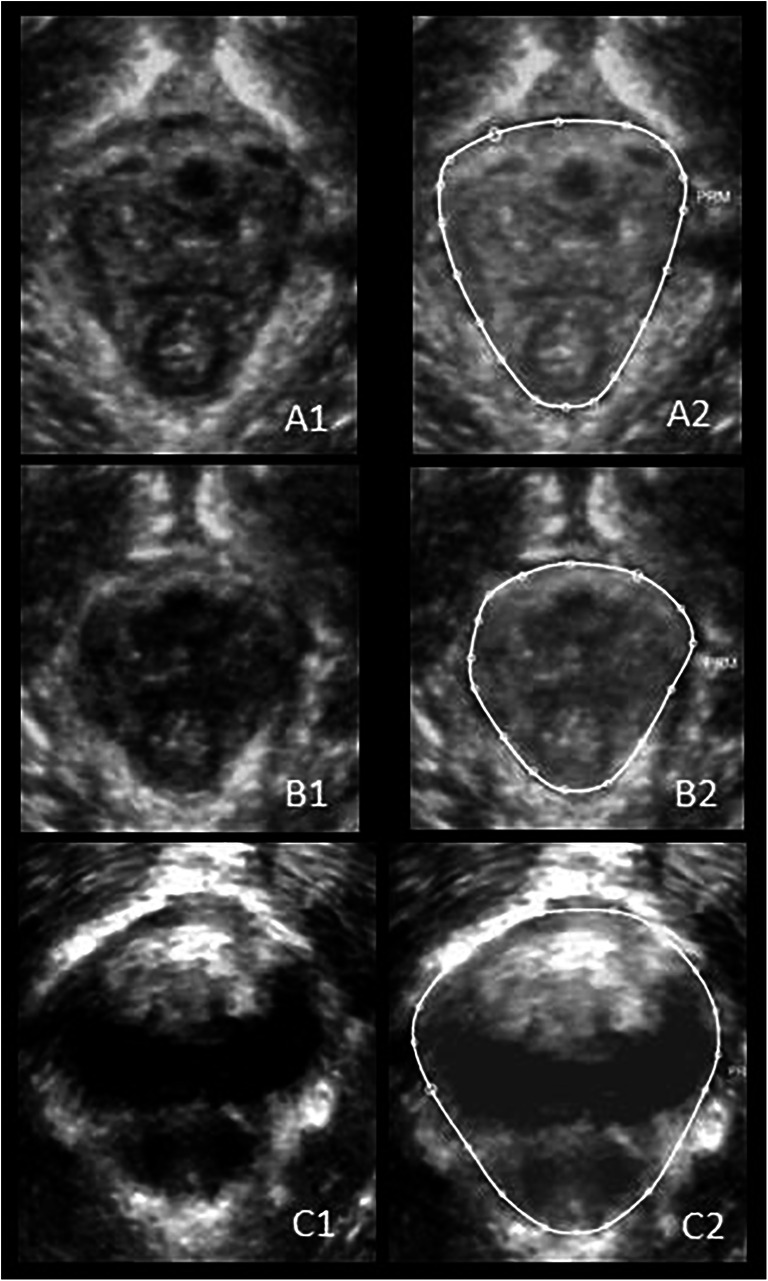
Hiatal areas without (*1*) and with (*2*) segmentation. **a** Hiatal area at rest (14.81 cm^2^). **b** Hiatal area at maximal contraction (12.05 cm^2^). **c** Hiatal area on maximal Valsalva (29.32 cm^2^)

**Fig. 3:**
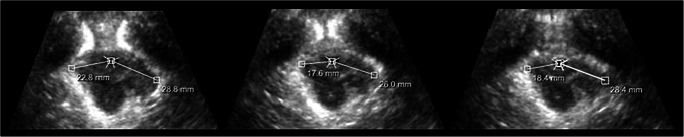
Transperineal ultrasound of a woman with complete unilateral avulsion showing the three central slices. On one side the levator–urethra gap is <25 mm (i.e., intact), whereas on the other side it is ≥25 mm (i.e., complete avulsion)
